# Postoperative shunt failure following hemispherectomy in pediatric patients with pre-existing hydrocephalus

**DOI:** 10.1007/s00381-024-06295-x

**Published:** 2024-01-25

**Authors:** Nikita Das, Akshay Sharma, Michael Mann, Alan Gordillo, Ansh Desai, Demitre Serletis, Ahsan N. Moosa, Richard Rammo, William Bingaman

**Affiliations:** 1 School of Medicine, Case Western Reserve University, Cleveland, OH USA; 2 Epilepsy Center, Cleveland Clinic Neurological Institute, Cleveland, OH USA; 3 Department of Neurological Surgery, Cleveland Clinic, Cleveland, OH USA

**Keywords:** Hydrocephalus, Drug resistant epilepsy, Hemispherectomy

## Abstract

**Objective:**

The risk of hydrocephalus following hemispherectomy for drug resistant epilepsy (DRE) remains high. Patients with pre-existing hydrocephalus pose a postoperative challenge, as maintaining existing shunt patency is necessary but lacks a clearly defined strategy. This study examines the incidence and predictors of shunt failure in pediatric hemispherectomy patients with pre-existing ventricular shunts.

**Methods:**

We performed a retrospective chart review at our center to identify pediatric patients diagnosed with DRE who were treated with ventricular shunt prior to their first hemispherectomy surgery. Demographic and perioperative data were obtained including shunt history, hydrocephalus etiology, epilepsy duration, surgical technique, and postoperative outcomes. Univariate analysis was performed using Fisher’s exact test and Pearson correlation, with Bonferroni correction to *a* = 0.00625 and *a* = 0.01, respectively.

**Results:**

Five of nineteen (26.3%) patients identified with ventriculoperitoneal shunting prior to hemispherectomy experienced postoperative shunt malfunction. All 5 of these patients underwent at least 1 shunt revision prior to hemispherectomy, with a significant association between pre- and post-hemispherectomy shunt revisions. There was no significant association between post-hemispherectomy shunt failure and valve type, intraoperative shunt alteration, postoperative external ventricular drain placement, hemispherectomy revision, lateralization of shunt relative to resection, postoperative complications, or postoperative aseptic meningitis. There was no significant correlation between number of post-hemispherectomy shunt revisions and age at shunt placement, age at hemispherectomy, epilepsy duration, or shunt duration prior to hemispherectomy.

**Conclusions:**

Earlier shunt revision surgery may portend a subsequent need for shunt revision following hemispherectomy. These findings may guide neurosurgeons in counseling patients with pre-existing ventricular shunts prior to hemispherectomy surgery.

**Supplementary Information:**

The online version contains supplementary material available at 10.1007/s00381-024-06295-x.

## Introduction

Since its advent in the 1920s, the use of hemispherectomy for the treatment of intractable epilepsy has evolved significantly due to advancements in anesthesia, neuroimaging, and microsurgical techniques [1–4]. Postoperative hydrocephalus has emerged as a well-documented sequela of hemispherectomy surgery [5–9]. Its etiology, though not fully understood, is attributed to surgical disruptions in cerebrospinal fluid (CSF) production-absorption patterns or CSF flow obstruction. The incidence of hydrocephalus following hemispherectomy has been estimated between 10 and 30% [5, 6, 8, 9]. The risk of developing post-hemispherectomy hydrocephalus depends on factors such as the underlying etiology that led to the hemispherectomy, the patient’s age, and the type of surgical approach used [2, 5–9]. Whereas some cases of postoperative hydrocephalus develop early on in the postoperative course, many patients develop hydrocephalus in a delayed fashion, months to years after the hemispherectomy procedure [1, 10–13].

Considering the evolving scientific theory of fluid dynamics within the central nervous system, it is clear that understanding the complex phenomenon of post-hemispherectomy hydrocephalus will require a more nuanced approach. Patients with pre-existing hydrocephalus pose a postoperative challenge, as maintaining existing shunt patency is necessary but lacks a clearly defined strategy. This investigation aims to identify predictive factors of postoperative shunt failure following hemispherectomy in pediatric patients with pre-existing ventriculoperitoneal shunts. The overarching goal of this case series is to provide guidance on counseling points that should be considered when discussing the risks and benefits of hemispherectomy surgery for pediatric patients with drug resistant epilepsy who have been previously treated for hydrocephalus.

## Methods

### Surgical and postoperative protocol

At our institution, the postoperative care routine has evolved over the last 25 years as performing hemispherectomy surgery has become a more prevalent treatment option for patients with drug resistant epilepsy (DRE). Functional hemispherectomy, also referred to as disconnective hemispherectomy in this series, incorporates frontal and parieto-occipital disconnection along with core temporal and central excision. Modified anatomic hemispherectomy involves a larger cortical resection of the pathological tissue with disconnection, and thus preservation, of the tissues that exhibit less epileptogenicity. At our institution, all hemispherectomy techniques involve opening of the ventricular system and cauterization of the portion of the choroid plexus near the Foramen of Monro that is visualized. A cottonoid is used to occlude the foramen as soon as it is exposed through the duration of the procedure to limit the passage of blood products into the contralateral ventricular system; however, it is not feasible to prevent blood products from collecting within the ventricular system on the side of surgery. Further details on our surgical technique are reported in our recent study on predictive factors of hydrocephalus following hemispherectomy in pediatric DRE patients [14].

Most patients undergoing the procedure will have a ventricular catheter placed intra-operatively within the resection cavity. If they have a pre-existing shunt with an adjustable valve, the shunt will be turned to its highest setting or “off,” in order to reduce the flow of bloody or proteinaceous surgical debris through the shunt during the perioperative period. Fixed-valve shunts are left unchanged. The ventricular catheter is clamped in the 24-h post-resection period to allow for reaccumulation of cerebrospinal fluid. A subgaleal drain is also placed at the time of surgery with suction turned on. The ventricular drain is opened to 10 mmHg above the tragus on postoperative day 1, and the subgaleal drain is taken off suction. On postoperative day 2, the subgaleal drain is removed and the ventricular drain is dropped until CSF drainage of 5–10 mL per hour is achieved. This is performed in order to remove blood and postoperative debris from the resection cavity and ventricles, possibly reducing the incidence of aseptic meningitis and shunt occlusion. The external ventricular drain (EVD) is typically removed between postoperative days 3 and 5, with the timing determined primarily by the color and quality of CSF output. The patient’s shunt is then returned to its original settings. Patients are managed in the Pediatric Intensive Care Unit until the ventricular drain is removed. While the EVD is in place, daily CSF samples are sent for routine culture analysis, collected directly from the distal collection column of the system by nursing staff, using a sterile technique. It is not uncommon for blood to be found in the ventricular system through postoperative CT imaging, though this parameter is difficult to quantify in a standardized fashion.

### Data collection

We performed a retrospective chart review at our center of all patients under 18 years of age with medically refractory epilepsy who were diagnosed with hydrocephalus and treated with ventricular shunt prior to undergoing hemispherectomy surgery between 1997 and 2016. Demographics, clinical features, and perioperative data were collected, including shunt location and history, etiology of hydrocephalus, epilepsy duration, age at hemispherectomy, surgical technique, and postoperative outcomes including complications (intracranial hemorrhage, perioperative stroke, or wound infection); aseptic meningitis (new fever of unknown origin with physical symptoms indicative of meningeal inflammation and a negative CSF bacterial culture); external ventricular drain (EVD) use; hemispherectomy revision surgery; post-hemispherectomy shunt revision; and Engel outcome at last follow-up.

### Data analysis

Univariate analysis was performed using Pearson’s correlation for continuous metric variables and Fisher’s exact test for categorical variables. Due to the high number of comparisons calculated, we opted to use the Bonferroni correction to minimize the risk of Type I errors [15]. The significance threshold for hypothesis testing on the nominal variables was determined as *a*_corrected_ = 0.00625. The significance threshold for correlation testing on the metric variables was determined as *a*_corrected_ = 0.01.

### Outcomes

Our primary outcome was the occurrence of post-hemispherectomy shunt failure, defined as the need for shunt revision or replacement following hemispherectomy surgery. Secondary outcomes included the time between hemispherectomy and shunt failure and the number of post-hemispherectomy shunt revisions.

## Results

### Demographics and characteristics of patient cohort (Table 1)

**Table 1:** Results summary

Patient cohort (*n* = 19)	
Gender	# (%) of patients
Male	13 (68.4%)
Female	6 (31.6%)
Race	# (%) of patients
Caucasian	17 (89.5%)
African American	2 (10.5%)
	Median age (years) [IQR]
Age at seizure onset	2.33 [0.25–3.21]
Age at shunt placement	0.42 [0.30–2.70]
Age at hemispherectomy surgery	9.08 [5.75–12.9]
Duration of seizures prior to hemispherectomy	5.70 [3.7–10.1]
Duration of shunt prior to hemispherectomy	6.20 [4.3–10.3]
Etiology of hydrocephalus	# (%) of patients
Congenital/idiopathic	3 (15.8%)
Trauma/TBI	6 (31.6%)
Perinatal vascular insult (hemorrhage or stroke)	7 (36.8%)
Tumor/neoplasm	3 (15.8%)
Type of hemispherectomy surgery	# (%) of patients
Functional	18 (94.7%)
Modified anatomic	1 (5.3%)
Prior resective neurosurgery	# (%) of patients
Yes	5 (26.3%)
No	14 (73.7%)
Side of shunt relative to hemispherectomy	# (%) of patients
Ipsilateral	8 (42.1%)
Contralateral	9 (47.4%)
Both ipsilateral and contralateral	2 (10.5%)
Location of proximal ventricular catheter	# (%) of patients
Frontal	8 (42.1%)
Parieto-occipital	11 (57.9%)
Temporal	0 (0%)
Location of distal ventricular catheter	# (%) of patients
Peritoneum	19 (100%)
Valve type prior to hemispherectomy	# (%) of patients
Fixed	14 (73.7%)
Adjustable	5 (26.3%)
# Patients with post-hemispherectomy shunt failure	5 (26.3%)
Median time to shunt failure post-hemispherectomy (years) [IQR]	0.42 [0.13–1.29]

Out of 296 pediatric patients who have undergone hemispherectomy at our institution between 1997 and 2016, 19 (6.4%) patients were identified who had an indwelling ventriculoperitoneal shunt prior to their first hemispherectomy procedure. Thirteen patients (68.4%) were male, and six patients (31.6%) were female. Seventeen patients (89.5%) were Caucasian, while two patients (10.5%) were African American. The median age at shunt placement was 0.42 years (IQR 0.3–2.7). The median age at seizure onset was 2.33 years (IQR 0.3–3.2). The median age of patients at hemispherectomy surgery was 9.1 years (IQR 5.8–12.9). The median duration of seizures prior to hemispherectomy surgery was 5.7 years (IQR 3.7–10.1), and the median duration of ventricular shunting prior to hemispherectomy surgery was 6.2 years (IQR 4.3–10.3). Three patients (15.8%) developed hydrocephalus attributed to congenital/idiopathic factors; six patients (31.6%) due to trauma or traumatic brain injury; seven patients (36.8%) from perinatal vascular insult (ischemic or hemorrhagic stroke); and three patients (15.8%) were associated with tumors/neoplasms. Eighteen patients (94.7%) underwent functional hemispherectomy procedures, and only one patient (5.3%) underwent a modified anatomic hemispherectomy (centrotemporal resection with exclusive anterior or posterior disconnection). Five patients (26.3%) had resective surgery (non-hemispherectomy) prior to their hemispherectomy. Eight patients (42.1%) had ipsilateral placement of their proximal shunt catheter relative to the side of hemispherectomy resection, while nine patients (47.4%) had contralateral placement. Two patients (10.5%) had bilateral ventriculoperitoneal shunts prior to hemispherectomy. Fourteen patients (73.7%) had a fixed pressure shunt valve in place at the time of hemispherectomy surgery, while five patients (26.3%) had an adjustable pressure valve in place prior to hemispherectomy. No shunts with anti-siphon devices were encountered. Four out of nineteen patients (21.1%) developed signs of infection within their postoperative course before discharge, and two out of five patients (40.0%) presenting with postoperative shunt failure developed infection within their postoperative course.

### Primary outcome: incidence of post-hemispherectomy shunt failure (Table 2)

**Table 2:** Primary outcome association tests-incidence of post-hemispherectomy shunt failure

**Categorical association tests**	**Fisher’s ** ***P***
Pre-hemispherectomy shunt revision and post-hemispherectomy shunt revision	0.005*
Post-hemispherectomy EVD placement and post-hemispherectomy shunt revision	0.603
Hemispherectomy revision and post-hemispherectomy shunt revision	0.058
Shunt valve type and post-hemispherectomy shunt revision	0.084
Ventricular shunt catheter placement relative to resection and post-hemispherectomy shunt revision	0.035
Intra-operative shunt alteration and post-hemispherectomy shunt revision	0.99
Postoperative aseptic meningitis and post-hemispherectomy shunt revision	0.53
Postoperative complications (ICH, perioperative stroke, wound infection) and post-hemispherectomy shunt revision	0.99

^*^Significant at *a*_corrected_ = 0.00625

**Fig. 1:**
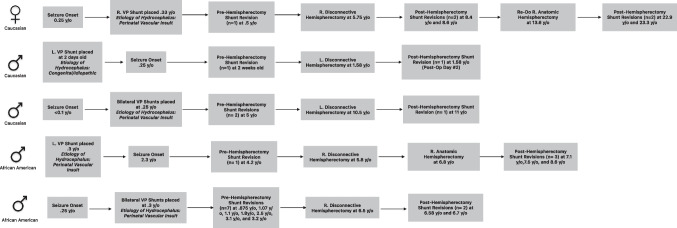
Individualized timelines of patients with post-hemispherectomy shunt failure

Five of nineteen patients in this sample (26.3%) required shunt revision due to shunt malfunction following hemispherectomy. The median time to post-hemispherectomy shunt failure within this sample was 0.42 years (IQR 0.125–1.29 years). The time to first post-hemispherectomy shunt revision ranged from 2 days to 2.6 years. All 5 patients underwent at least 1 shunt revision prior to hemispherectomy, with a statistically significant association between the occurrence of pre- and post-hemispherectomy shunt revision (*p* = 0.005). There was no association between post-hemispherectomy shunt failure and valve type (*p* = 0.084); intraoperative shunt alteration (*p* = 0.99); postoperative external ventricular drain (EVD) use (*p* = 0.603); hemispherectomy revision surgery (*p* = 0.058); location of ventricular shunt catheter relative to resection (*p* = 0.035); occurrence of postoperative complications (*p* = 0.99); or postoperative aseptic meningitis (*p* = 0.53). There was a roughly equivalent distribution of patients who experienced early postoperative shunt failure within 60 days post-hemispherectomy (*n* = 2) and patients who presented with delayed postoperative shunt failure occurring > 60 days postoperatively (*n* = 3). Those who experienced late-onset shunt failure following hemispherectomy generally were older at the time of hemispherectomy (median age at time of surgery: 5.75 years, IQR: 5.8–8.1) in comparison to patients who experienced early-onset shunt failure following hemispherectomy (median age at time of surgery: 4.02 years, IQR: 2.8–5.2). A two-sample *t*-test suggested this difference is not statistically significant (*p* = 0.31). Additionally, patients with delayed onset of postoperative hydrocephalus typically had shunts for a longer duration prior to hemispherectomy than those presenting with early onset postoperative hydrocephalus (median shunt duration: 5.4, IQR: 5.4–7.9 vs. vs. 4.02, IQR 2.8–5.2 years, respectively). A two-sample *t*-test found this difference not to be statistically significant (*p* = 0.32). The etiology of hydrocephalus for these patients presenting with postoperative shunt failure was either congenital/idiopathic (*n* = 1) or attributed to perinatal vascular insult (*n* = 4) (Fig. 1).

### Secondary outcomes: timing to and number of post-hemispherectomy shunt revisions

Concerning the metric variables obtained from the data, Pearson correlation testing revealed no statistical association between the time to first post-hemispherectomy shunt failure and age at shunt placement (*r* = 0.66, *p* = 0.107); age at hemispherectomy surgery (*r* = 0.24, *p* = 0.603); epilepsy duration (*r* = −0.03, *p* = 0.957); shunt duration prior to hemispherectomy (*r* = 0.25, *p* = 0.592); or number of pre-hemispherectomy shunt revisions (*r* = −0.45, *p* = 0.311). Additional Pearson correlation analyses revealed no statistically significant correlation between the number of post-hemispherectomy shunt revisions required and the age at shunt placement (*r* = −0.28, *p* = 0.252), age at the time of hemispherectomy surgery (*r* = −0.35, *p* = 0.137), epilepsy duration (*r* = −0.2, *p* = 0.402), or shunt duration prior to hemispherectomy (*r* = −0.54, *p* = 0.211). There is a medium, positive correlation between the number of pre-hemispherectomy shunt revisions and the number of post-hemispherectomy shunt revisions in this sample (*r* = 0.43, *p* = 0.069). This association did not reach statistical significance.

## Discussion

Hydrocephalus is widely acknowledged as a potential complication of hemispherectomy surgery, yet there remains ambiguity regarding its underlying risk factors and mechanisms of onset. There is a scarcity of literature exploring this phenomenon in pediatric hemispherectomy patients previously treated for hydrocephalus, who are often excluded from hemispherectomy study cohorts in efforts to minimize confounding [5]. Though limited by a relatively small sample size (*n* = 19), this study provides insight into the factors associated with shunt malfunction following hemispherectomy in pediatric patients with pre-existing ventriculoperitoneal shunts.

Out of 296 hemispherectomy patients, 19 patients (6.4%) were diagnosed and treated for pre-existing hydrocephalus prior to undergoing hemispherectomy. The median duration of shunting prior to hemispherectomy was approximately 6 years (Table 1). The majority of the procedures in this series were functional hemispherectomies (94.7%), whereas only one patient included in this examination (5.3%) underwent an anatomic hemispherectomy (Table 1). A total of 26.3% of patients in this study required shunt revision due to shunt malfunction following hemispherectomy, with the median time to shunt failure as approximately 5 months. The most common reason for shunt failure was proximal catheter obstruction. Other reasons for shunt revision in this series included valve dysfunction and distal catheter infection. Whereas we previously found the infection rate of pediatric patients undergoing hemispherectomy to be 10% [14], this study shows that patients with pre-existing ventriculoperitoneal shunts have an increased likelihood of postoperative infection following hemispherectomy, at approximately 20%. It is possible that postoperative infection increases the likelihood of shunt failure following hemispherectomy, considering that two of the five patients who experienced postoperative shunt failure also had postoperative infection. The limited sample size of this cohort precludes meaningful statistical analysis of this relationship.

Our data supports that patients who experience shunt malfunction prior to hemispherectomy surgery are more likely to require postoperative shunt revision. Importantly, our findings herein suggest that the use of postoperative EVD does not appear to be associated with any higher shunt complication rate in hemispherectomy patients with pre-existing hydrocephalus. The positive association between pre-hemispherectomy shunt revisions and post-hemispherectomy shunt revisions suggests that the incidence of previous shunt revision surgery may be prognostic of poor postoperative shunt patency. Full interpretation of this trend is limited when considering that certain subpopulations of patients with pre-existing hydrocephalus have a greater underlying susceptibility to shunt malfunction throughout their lifetime, dependent on many different factors [12, 16].

Within our series, the time to first post-hemispherectomy shunt revision ranged from 2 days to 2.6 years, supporting existing consensus that hydrocephalus can occur relatively soon after surgery, or may take a more latent course of development [2, 5–9]. Regarding patients who experienced shunt failure > 60 days postoperatively, it is possible that the hemispherectomy had little or no bearing on the episode of delayed shunt malfunction. Of the three patients presenting with delayed post-hemispherectomy hydrocephalus, one patient presented with symptoms of postoperative hydrocephalus requiring intervention 5 months postoperatively, while the other two patients required postoperative shunt revision 1.3 and 2.6 years post-hemispherectomy. It is conceivable that patients presenting with shunt failure within 1 year postoperatively are more likely to have hydrocephalus specifically attributed to the surgical intervention, whereas those presenting with shunt failure years after surgery could be due to factors unrelated to the hemispherectomy procedure. Nonetheless, any neurosurgical procedure can lead to scarring and fibrosis of the region of interest, which may impede the normal drainage pathways of CSF, leading to a delayed-onset hydrocephalus [17]; this could explain the mechanism underlying delayed onset postoperative hydrocephalus. Still, the pathophysiological differences of early versus delayed onset postoperative hydrocephalus have yet to be discerned.

The etiology of hydrocephalus likely influences a patient’s propensity to develop shunt failure. In our sample, the etiology of hydrocephalus for the children who presented with postoperative shunt failure was congenital/idiopathic (*n* = 1) or due to perinatal vascular insult (*n* = 4). Existing literature implicates the etiology of hydrocephalus, particularly intraventricular hemorrhage and congenital defects, as potential risk factors for shunt failure [8, 18]. Patients with a history of perinatal intracranial hemorrhage might exhibit a baseline pattern of proinflammatory expression causing ependymal damage, predisposing them to an increased risk for acquired postoperative hydrocephalus [5, 19]. This perspective provides a conceptual framework through which to interpret the positive association between the frequency of preoperative shunt revisions and post-hemispherectomy shunt failure. Still, the pathophysiological mechanisms leading to the onset of postoperative hydrocephalus remain unclear. The inflammatory component predisposing to hydrocephalus may be linked to the buildup of pro-inflammatory molecules within the brain’s interstitial fluid and ventricular system [20]. If the efflux mechanisms of these molecules are impaired or overwhelmed, then the increased osmolarity of the interstitial fluid may draw water into the ventricular system, leading to hydrocephalus [20]. Clearance of inflammatory debris from the brain interstitium is a process dependent on the expression of efflux transporters such as p-glycoprotein along the Blood Brain Barrier [20]. This is the basis for current investigations examining whether perioperative steroids—such as dexamethasone, a known inducer of p-glycoprotein [20]—may be useful in prophylaxis of postoperative hydrocephalus.

Other pertinent considerations regarding risk factors for shunt failure include shunt location and valve type. Previous investigations report conflicting findings on whether the shunt approach affects shunt revision rates. In 2009, a prospective study performed by Farahmand et al. found that placement of the proximal ventricular catheter in the right frontal lobe was associated with a lower rate of shunt revision within 6 months of insertion in adult patients with hydrocephalus [11] Contrarily, in 2019, Bhargav et al. reported negligible difference in revision rates between frontal and parietal approaches for ventricular shunt placement in the treatment of idiopathic normal pressure hydrocephalus [21]. Our study does not provide convincing evidence of a skewed distribution of shunt failure depending on the location of the proximal catheter, given that there is a relatively balanced distribution of frontal (*n* = 2) and parieto-occipital (*n* = 3) proximal catheter placements for patients presenting with post-hemispherectomy shunt failure. In this study, two of five patients requiring post-hemispherectomy shunt revision had ipsilateral placement of proximal shunt catheter, one of five patients had contralateral placement, and two of five patients had bilateral shunts. At the significance threshold of *a* = 0.00625, our data shows no significant association linking the ipsilateral location of the ventricular shunt catheter to a higher incidence of post-hemispherectomy shunt failure (*p* = 0.035) (see “Limitations”). Additionally, of the patients in this sample who developed postoperative hydrocephalus, two had fixed pressure valves in place prior to hemispherectomy, and three had adjustable pressure valves. Whereas some studies suggest adjustable valves may lessen the risk for shunt failure [11], others have found no association between valve programmability and shunt patency [18, 22]. Further investigation is needed in order to discern the true relationship between valve type and risk for postoperative hydrocephalus.

Meanwhile, our study reports no statistically significant association between post-hemispherectomy shunt failure and intraoperative shunt alteration; postoperative EVD usage; postoperative complication; or postoperative aseptic meningitis. This implies that postoperative management of pediatric hemispherectomy patients with pre-existing ventriculoperitoneal shunts should be dichotomized into consideration of (i) factors affecting acute perioperative care and (ii) factors affecting longitudinal shunt patency.

### Limitations

This study has several limitations. The retrospective design serves as an intrinsic constraint, primarily due to the nature of electronic medical record (EMR) data collection, which may be subject to recall bias, incomplete documentation, or changes in data collection practices over time. We attempted to mitigate analysis errors attributed to these factors by reviewing all note types in each patient’s EMR, repeating chart review for all patients three times, and cross-referencing extracted data among the authors responsible for data collection. Additionally, the generalizability of this study is confined by its small sample size. The limited number of hemispherectomy cases performed by any single epilepsy center has restricted the availability of data on risk factors for post-hemispherectomy complications. Nonetheless, the benefit conferred by case studies is the detail of patient data, which we seek to provide with transparency in order to add to the growing fund of literature exploring hydrocephalus as a postoperative sequela of hemispherectomy surgery (Online Resource 1: Supplemental Table).

Due to the small sample size, the statistical tests performed on our data may not have sufficient power to detect a statistically significant difference, even if a clinically meaningful difference exists. It is worth noting that an appreciable critique of the Bonferroni correction is that it is overly conservative; its use decreases the risk of Type I errors, with a risk of increasing Type II errors and potentially missing valid significant findings [15]. Considering that the historical determination of the standard *a* = 0.05 has been set arbitrarily, it may be important to pay attention in clinical settings and future studies to variables nearing statistical significance *before* the Bonferroni adjustment—such as the number of pre-hemispherectomy shunt revisions, valve type, need for hemispherectomy revision surgery, or lateralization of proximal shunt catheter relative to resection. Ultimately, the conclusions to be drawn from the statistics reported in this case series should be interpreted within the context of existing literature exploring risk factors for postoperative hydrocephalus.

### Relevance and future applications

The intent of this case series is to draw attention to certain clinical features in the past medical history that should be reviewed by neurosurgeons, neurologists, and other clinical team members when consulting families about postoperative expectations and risks following hemispherectomy in patients with pre-existing ventriculoperitoneal shunts. The frequency of shunt revisions prior to hemispherectomy may be indicative of a patient’s propensity to develop post-hemispherectomy hydrocephalus necessitating further shunt revision. A multicenter prospective study would be helpful to corroborate the findings of this case series and further distinguish risk factors for postoperative hydrocephalus in pediatric hemispherectomy patients with previously shunted hydrocephalus.

## Conclusions

In conclusion, the incidence of previous shunt revisions is associated with post-hemispherectomy shunt failure in pediatric patients with existing shunted hydrocephalus. The occurrence of at least one pre-hemispherectomy shunt revision is related to an increased risk for post-hemispherectomy shunt failure. Incidence of prior shunt revision surgery should therefore be regarded as a clinical proxy for a given patient’s susceptibility to developing postoperative hydrocephalus following hemispherectomy surgery.

### Supplementary Information

Below is the link to the electronic supplementary material.

Supplementary file1 (PDF 144 KB)
