# Supplementary material for: Postoperative shunt failure following hemispherectomy in pediatric patients with pre-existing hydrocephalus
Source: Childs Nerv Syst. 2024 Jan 25;40(5):1507–14. doi: 10.1007/s00381-024-06295-x (PMC11026181; doi:10.1007/s00381-024-06295-x)
Supplement: Supplementary file 1 — Supplementary file1 (PDF 144 KB) [file 381_2024_6295_MOESM1_ESM.pdf]

Title:

Postoperative Shunt Failure Following Hemispherectomy in Pediatric Patients with Pre-Existing Hydrocephalus

Authors:

Nikita Das[2], BA, Akshay Sharma[1], MD, Michael Mann[2], BS, Alan Gordillo[2], BS, Ansh Desai[2], BA, Demitre Serletis[1], MD, PhD, FRCSC, Ahsan N Moosa[1], MD, Richard Rammo[1], MD, and William Bingaman[1], MD

Affiliations:

[1]Epilepsy Center, Cleveland Clinic Neurological Institute, Cleveland, OH, USA
[2]School of Medicine, Case Western Reserve University, Cleveland, OH, USA

Corresponding Author's name and current institution:

Akshay Sharma, Cleveland Clinic Department of Neurological Surgery

Corresponding Author's Email:

sharmaa5@ccf.org

Keywords:

Hydrocephalus, Drug Resistant Epilepsy, Hemispherectomy

Supplemental Table: Demographics & Characteristics of Patient Sample

PT	Gender	Race	Etiology of Hydrocephalus	Age at Seizure Onset (yrs)	Age at Shunt Placement (yrs)	Type of Shunt	Age at Hemispherectomy (yrs)	Type of Hemispherectomy	Prior Resective Surgery	Intra-Operative Shunt Alteration	Post-Op EVD	Need for Shunt Revision Post-Hemispherectomy	Need for Hemispherectomy Revision Surgery	Engel Outcome
#1	Male	Caucasian	Trauma/ TBI	3.42	4.00	R. Occipital Ventriculoperitoneal	9.08	L. Disconnective	No	No	No	No	No	Engel I
#2	Male	Caucasian	Perinatal Vascular Insult	0	0.92	L. Frontal Ventriculoperitoneal	16.58	L. Disconnective	No	No	No	No	No	Engel I
#3	Female	Caucasian	Tumor/ Neoplasm	1.5	1.33	L. Frontal Ventriculoperitoneal	5.83	R. Disconnective	Yes	No	No	No	No	Engel I
#4	Female	Caucasian	Tumor/ Neoplasm	3	0.83	L. Parietal Ventriculoperitoneal	12.83	R. Disconnective	Yes	No	No	No	No	Engel I
#5	Male	Caucasian	Congenital/ Idiopathic	0.25	0	L. Frontal Ventriculoperitoneal	1.58	L. Disconnective	No	No	No	Yes	No	Engel III-IV
#6	Male	Caucasian	Perinatal Vascular Insult	0	0.25	R. Parietal & L. Occipital Ventriculoperitoneal	10.50	L. Disconnective	No	No	Yes	Yes	No	Engel I
#7	Male	Caucasian	Congenital/Idiopathic	0	0.33	L. Parietal Ventriculoperitoneal	2.17	L. Disconnective	No	No	Yes	No	No	Engel I
#8	Female	Caucasian	Perinatal Vascular Insult	0.25	0.33	R. Parietal Ventriculoperitoneal	5.75	R. Disconnective	No	No	Yes	Yes	Yes (Anatomic Hemispherectomy 7.8 years after Functional Hemispherectomy)	Engel III-IV
#9	Female	Caucasian	Trauma/ TBI	3	16.58	R. Frontal Ventriculoperitoneal	18.04	R. Disconnective	Yes	No	Yes	No	No	Engel III-IV
#10	Female	Caucasian	Trauma/ TBI	5.5	5.50	R. Parietal Ventriculoperitoneal	13.42	L. Disconnective	No	No	Yes	No	No	Engel III-IV
#11	Female	Caucasian	Perinatal Vascular Insult	2.5	0.42	R. Frontal Ventriculoperitoneal	4.50	R. Disconnective	No	No	Yes	No	No	Engel II
#12	Male	Caucasian	Tumor/Neoplasm	2.5	1.13	R. Frontal Ventriculoperitoneal	3.13	L. Modified Anatomic	Yes	No	Yes	No	No	Engel I
#13	Male	African American	Perinatal Vascular Insult	2.33	0.33	L. Frontal Ventricu	5.75	R. Disconnective	No	No	Yes	Yes	Yes (Anatomic Hemispherectomy 1.1	Engel I

						loperitoneal							years after Functional Hemispherectomy)	
#14	Male	Caucasian	Congenital/Idiopathic	0	0.25	L. Occipital Ventriculoperitoneal	11.90	L. Disconnective	No	No	Yes	No	No	Engel I
#15	Male	Caucasian	Perinatal Vascular Insult	4.29	0	R. Parietal Ventriculoperitoneal	8.33	L. Disconnective	No	No	Yes	No	No	Engel I
#16	Male	Caucasian	Trauma / TBI	8.5	7.50	L. Occipital Ventriculoperitoneal	12.67	L. Disconnective	No	Yes (shunt switched from the left ventricle to right lateral ventricle)	Yes	No	No	Not Available
#17	Male	Caucasian	Trauma / TBI	9	7.50	L. Parietal Ventriculoperitoneal	17.67	R. Disconnective	Yes	No	No	No	No	Engel I
#18	Male	Caucasian	Trauma / TBI	0.29	0.30	R. Frontal Ventriculoperitoneal	12.98	L. Disconnective	No	No	No	No	No	Engel III-IV
#19	Male	African American	Perinatal Vascular Insult	0.25	0.31	L. Parietal & R. Occipital Ventriculoperitoneal	6.46	R. Disconnective	No	No	Yes	Yes	No	Not Available
